# Comparing the Impact of Flooding on Mental Health in India and the United Kingdom: Who Is More Vulnerable?

**DOI:** 10.1192/bjo.2025.10109

**Published:** 2025-06-20

**Authors:** Peter Carter, Chartlotte Opoku Gyamfi, Tasneem Quddus, Mershid Varnasseri Ghandali, Ellie Hawken

**Affiliations:** Anglia Ruskin University, Chelmsford, United Kingdom

## Abstract

**Aims:** We hypothesise that mental health impacts of flooding will be greater in India compared with the UK. Climate change is causing an increase in flooding due to the rising frequency of extreme weather patterns globally. The major impacts of flooding on mental health include displacement, financial hardship, and loss of access to healthcare. These can lead to conditions such as anxiety, depression, and post-traumatic stress disorder (PTSD).

**Methods:** This study was conducted as a comparative analysis. Data was collected by a systematic search of peer-reviewed articles. Standardised tools were used to evaluate psychological outcome and mental health morbidities such as the Generalised Anxiety Disorder scale (GAD-7), Patient Health Questionnaire (PHQ-9), WHO-5 and the PTSD checklist (PCL-6). Data concerning the mental health consequences caused by the floods (specifically regarding anxiety, depression and PTSD), financial impacts and access to mental health services in both countries were extracted. Our findings were then thematically analysed to compare the patterns and disparities.

**Results:** In both countries, the research conducted on the effects of flooding on mental health has identified that the three main mental health morbidities that arose are depression, anxiety and PTSD. India has an average percentage of 43.2% depression, 32.19% anxiety and 36.46% PTSD amongst individuals affected by flooding, while the UK shows equivalent rates of 25.52% depression, 24.2% anxiety and 31.49% PTSD. These results suggest that socioeconomic differences and access to mental health resources play a significant role in post-flood psychological states. In both countries a larger financial impact links to higher rates of psychological stress.

**Conclusion:** Although effects are noted in both the UK and India, the prevalence of mental health conditions arising from flooding affect both the UK and India. However, our findings indicate that the mental health impacts are more severe in India, supporting our hypothesis. In disaster recovery, mental health funding is frequently deprioritised in favour of immediate concerns such as physical health and infrastructure.

Stigma surrounding mental health, particularly affecting developing countries, contributes to under-reporting and therefore the accuracy of assessments. To improve outcomes, a public health approach may destigmatise mental health, and enhance social support. Additionally, Psychological First Aid has set international foundations for psychosocial care following distressing events, a framework which supports people whilst respecting culture and abilities.

